# Inverse Problem for Color Doppler Ultrasound-Assisted Intracardiac Blood Flow Imaging

**DOI:** 10.1155/2016/6371078

**Published:** 2016-05-22

**Authors:** Jaeseong Jang, Chi Young Ahn, Jung-Il Choi, Jin Keun Seo

**Affiliations:** ^1^Department of Computational Science and Engineering, Yonsei University, 50 Yonsei-ro, Seodaemun-gu, Seoul 03722, Republic of Korea; ^2^Division of Integrated Mathematics, National Institute for Mathematical Sciences, 70 Yuseong-daero 1689 beon-gil, Yuseong-gu, Daejeon 34047, Republic of Korea

## Abstract

For the assessment of the left ventricle (LV), echocardiography has been widely used to visualize and quantify geometrical variations of LV. However, echocardiographic image itself is not sufficient to describe a swirling pattern which is a characteristic blood flow pattern inside LV without any treatment on the image. We propose a mathematical framework based on an inverse problem for three-dimensional (3D) LV blood flow reconstruction. The reconstruction model combines the incompressible Navier-Stokes equations with one-direction velocity component of the synthetic flow data (or color Doppler data) from the forward simulation (or measurement). Moreover, time-varying LV boundaries are extracted from the intensity data to determine boundary conditions of the reconstruction model. Forward simulations of intracardiac blood flow are performed using a fluid-structure interaction model in order to obtain synthetic flow data. The proposed model significantly reduces the local and global errors of the reconstructed flow fields. We demonstrate the feasibility and potential usefulness of the proposed reconstruction model in predicting dynamic swirling patterns inside the LV over a cardiac cycle.

## 1. Introduction

Vortex flow imaging has recently attracted much attention owing to a strong correlation between intraventricular flow pattern and heart function [1–3]. Possible clinical indices of cardiac functions can be obtained by characterizing and quantifying the vorticity of intraventricular blood flow. There are several studies to compute and quantify the blood flow pattern inside the left ventricle (LV) by using ultrasound imaging scanner. Echo particle image velocimetry (E-PIV) is representative of the commonly used method [4, 5], which tracks the speckle pattern of blood flow. However, the E-PIV is hardly a complete noninvasive method because it requires the intravenous injection of contrast agent.

As an alternative approach to reconstruct the velocity of blood flow, color flow ultrasound-based techniques have been proposed. Color flow ultrasound is also called C-mode images, color Doppler images, color Doppler data, or color Doppler ultrasound. Color flow images reflect the velocity components projected on the direction of ultrasound beam propagation [6]. They are represented as the colors of red and blue in a region of interest (ROI) box overlapped on echo images. In general, red and blue colors indicate the velocity components coming toward and receding from scanning probe, respectively. Among the color flow ultrasound-based techniques, one method is to reconstruct blood flow by using the assumption of 2D divergence-free blood flow [7, 8]. It decomposes each 2D velocity vectors into radial component obtained from color flow data and unknown angular component and computes the unknown component of blood flow velocity under the 2D divergence-free condition. However, it has limitation of oversimplification that ignores out-of-plane flows. Another method is to use the beams of multiple directions of color Doppler ultrasound. It reconstructs the 2D or 3D velocities of blood flow by using color Doppler data acquired from the beams in two or three different directions [9, 10]. However, the registration of multiple imaging planes by the multiple directional beams is a very challenging issue in practical environment. Recently, we proposed a 2D incompressible Navier-Stokes model to reconstruct intraventricular flows using color Doppler data and LV boundaries extracted from echocardiography data [11]. The model was designed to cope with out-of-plane blood flows for the imaging plane by introducing a mass source term of a source-sink distribution. Although the 2D model seemed to preserve the global kinetic energy and vortex strength during cardiac cycle, the predicted velocity and vorticity fields indicated that the model did not precisely capture the location and shape of dynamic vortex patterns.

**Figure 1:**
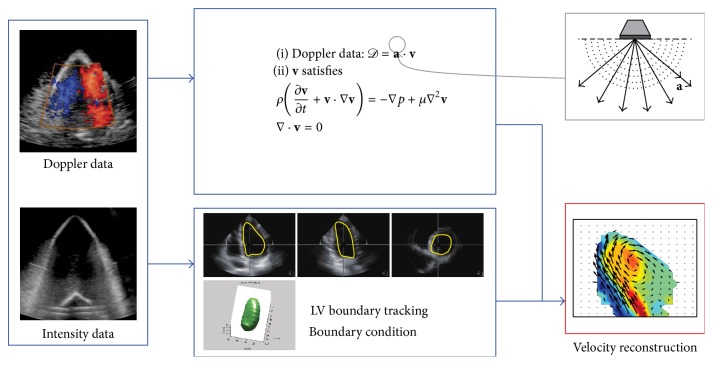
Overall framework for LV blood flow reconstructions based on echocardiographic images.

In this paper, we propose a mathematical framework in Figure 1 for reconstructing the blood flow in LV using 3D echocardiographic images and the color Doppler intensity data. The framework includes a formulation of inverse problem for undetermined velocity and pressure fields, LV boundary tracking, and a forward simulation procedure to generate synthetic flow data. Under the assumption that the high frame-rate acquisition of 3D echocardiographic data is available in the whole LV region, the color Doppler data is directly embedded in the incompressible Navier-Stokes equations, along with 3D LV boundary reconstruction. An interpolation technique using multiple planar LV contours extracted from the echocardiographic images is applied to obtain the boundary conditions on the 3D LV boundaries. We perform the forward simulation with the LV boundary data from real intensity images. Based on the simulation results, one-directional velocity component of the flow data is obtained for synthetic color Doppler data. Using the synthetic data, intracardiac blood flows inside LV are reconstructed by solving the inverse model. Finally, we demonstrate the robustness of the proposed model in visualizing time-dependent vortex patterns over one cardiac cycle and quantifying local and global errors for the reconstructed flow fields.

## 2. Methods

### 2.1. Problem Statement

**Figure 2:**
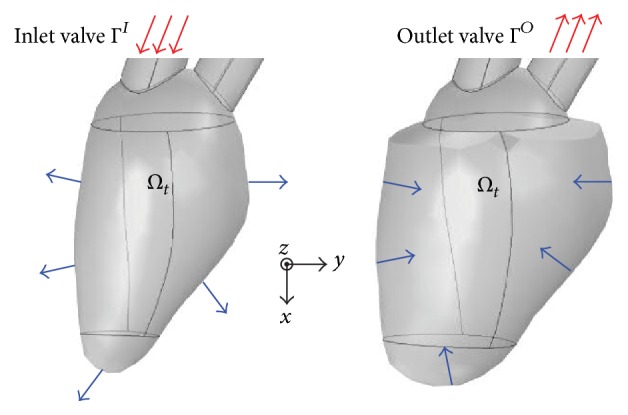
Description of 3D LV domain (*Ω*_*t*_) and the coordinate system. The arrows indicate the LV wall movements and flow directions over the inlet and outlet valves at the diastole and systole phases.

This section describes a mathematical framework of inverse problem of recovering the velocity distribution in LV by using color Doppler data. Let **v**(**x**, *t*) denote the velocity of blood flow at position **x** and time *t*, and let *Ω*_*t*_ be a 3D LV region at time *t*, as shown in Figure 2. Assuming that blood flow is an incompressible Newtonian fluid, the velocity **v** is governed by the incompressible Navier-Stokes equations inside the time-varying LV region *Ω*_*t*_:

(1) ∂v∂t+v·∇v=−1ρ∇p+μρ∇2vin  Ωt,∇·v=0in  Ωt,

where *ρ*, *μ*, and *p* are the density, viscosity, and pressure of the blood, respectively. To determine **v** in (1) uniquely, we need to impose proper boundary conditions of LV wall motion involving the inlet and outlet conditions over the inlet and outlet valves that are denoted by Γ^*I*^ and Γ^*O*^ in Figure 2, respectively. Since the velocity **v** obtained by solving (1) is sensitive to boundary conditions, it is very important to extract accurate LV wall motion. However, it is almost impossible to capture 3D LV wall motion accurately in practical environment. Therefore, supplementary information of **v** is necessary for computing blood flow reliably.

In this paper, we use the color Doppler data *𝒟*, as the additional information of one component of velocity, of the form

(2) $$\begin{array}{c} D=a\cdot v, \end{array}$$

where **a** is the ultrasound beam direction. Based on (1) and (2), our reconstruction model assimilates velocity components with the additional information. Details of the proposed reconstruction model are explained in the following section.

### 2.2. Reconstruction Model

We assume that the color Doppler data provides the first component of velocity (to be precise, $a={\left[\begin{array}{ccc} 1 & 0 & 0 \end{array}\right]}^{T}$). Using the inner product of **a** and the momentum equation in (1) with (2), we have

(3) ∂∂tD+D,v2,v3·∇D=μρ∇2D−1ρ∂∂xpin  Ωt,∂∂yv2+∂∂zv3=−∂∂xDin  Ωt.

The above system of equations can be expressed as the following matrix form:

(4) ∂D∂y∂D∂z∂∂y∂∂zv2v3=−∂D∂xD−∂∂tD+μρ∇2D−∂∂xD−1ρ∂∂xp0in  Ωt,

where the relation between *p* and **v** is given by

(5) $$\begin{array}{c} \frac{1}{\rho }\nabla^{2}p=-\nabla \cdot \left(\;v\cdot \nabla v\;\right)\;in\;\;\Omega_{t}. \end{array}$$

**Remark 1 .**

According to (4), if it is possible to measure subsidiary Doppler data *𝒟*_2_ along a direction **a**_2_ ≠ **a**, the velocity **v** can be explicitly expressed by

(6) v2v3=∂D∂y∂D∂z∂D2∂y∂D2∂z−1·−∂D∂xD−∂∂tD+μρ∇2D−∂D2∂xD−∂∂tD2+μρ∇2D2−1ρ∂∂xpa2·∇p.

Hence, if two pieces of linearly independent Doppler data are available, then the inverse problem can be simply solved without the information of LV boundary ∂*Ω*_*t*_.

To solve the velocity components *v*_2_ and *v*_3_ that satisfy (4), we consider an iterative procedure as follows: for the *n*th iteration, we find *v*_2_^*n*+1^ and *v*_3_^*n*+1^ such that

(7) ∂D∂y∂D∂z∂∂y∂∂zv2n+1v3n+1=−∂D∂xD−∂∂tD+μρ∇2D−∂∂xD−1ρ∂∂xpn+10in  Ωt,

where **v** ^*n*^ = (*𝒟*, *v*_2_^*n*^, *v*_3_^*n*^) and *p* ^*n*+1^ satisfy

(8) $$\begin{array}{c} \frac{1}{\rho }\nabla^{2}p^{n+1}=-\nabla \cdot \left(\;v^{n}\cdot \nabla v^{n}\;\right)\;in\;\;\Omega_{t}. \end{array}$$

Note that proper boundary condition is needed to guarantee the uniqueness of *p* ^*n*^ in (8). To describe boundary conditions for *v*_2_^*n*+1^, *v*_3_^*n*+1^, and *p* ^*n*+1^, we first simplify LV boundary near the valves by considering linearly connected parts to the valves in the LV domain. Since it is still challenging to accurately capture the valve geometry in B-mode images, we focus on the LV wall motion, while we ignore the motion of valves in this paper. Then, we impose proper boundary conditions on the simplified boundary. Considering the simplified LV domain *Ω*_*t*_′, the corresponding boundaries near valves and physical LV wall are defined as Γ_*t*_^*u*^ and Γ_*t*_^*w*^ = ∂*Ω*_*t*_′∖Γ_*t*_^*u*^, respectively. Thus, the proposed reconstruction model can be rewritten as,

(9) ∂D∂y∂D∂z∂∂y∂∂zv2n+1v3n+1=−∂D∂xD−∂∂tD+μρ∇2D−∂∂xD−1ρ∂∂xpn+10in  Ωt′,

with the boundary conditions

(10) v2n+1=v2wall,v3n+1=v3wallon  Γtw,∂v2n+1∂n=0,∂v3n+1∂n=0on  Γtu,

where **v** ^*n*^ and *p* ^*n*+1^ satisfy

(11) 1ρ∇2pn+1=−∇·vn·∇vnin  Ωt′,∂p∂n=0on  ∂Ωt′.

Here, the velocity components *v*_2_^wall^ and *v*_3_^wall^ are estimated from LV boundary segmentation. Moreover, the Neumann boundary condition is imposed to allow the reconstructed flow to pass through Γ_*t*_^*w*^.

The 3D domain is discretized by *N*_*x*_ × *N*_*y*_ × *N*_*z*_ node points, where *N*_*x*_, *N*_*y*_, and *N*_*z*_ are the numbers of nodes along *x*, *y*, and *z* directions, respectively, with uniform grid size *h*. we define discrete differential operators based on the finite difference method as follows: *𝔻*_*x*_ = *𝕀*_*N*_*z*__ ⊗ *𝕀*_*N*_*y*__ ⊗ *𝔻*_*N*_*x*__, *𝔻*_*y*_ = *𝕀*_*N*_*z*__ ⊗ *𝔻*_*N*_*y*__ ⊗ *𝕀*_*N*_*x*__, *𝔻*_*z*_ = *𝔻*_*N*_*z*__ ⊗ *𝕀*_*N*_*y*__ ⊗ *𝕀*_*N*_*x*__, and *𝕃* = (*𝕃*_*N*_*z*__ ⊗ *𝕀*_*N*_*y*__ ⊗ *𝕀*_*N*_*x*__)+(*𝕀*_*N*_*z*__ ⊗ *𝕃*_*N*_*y*__ ⊗ *𝕀*_*N*_*x*__)+(*𝕀*_*N*_*z*__ ⊗ *𝕀*_*N*_*y*__ ⊗ *𝕃*_*N*_*x*__), where ⊗ is the Kronecker product and

(12) Dn=012h−12h012h⋱−12h0n×n,Ln=−2h21h21h2−2h21h2⋱1h2−2h2n×n,In=10010⋱01n×n.

Using the above discrete operators, the following discrete system for **v**_2_^*n*^ = [*v*_2(1,1,1)_,…, *v*_2(*N*_*x*_,*N*_*y*_,*N*_*z*_)_]^*T*^, **v**_3_^*n*^ = [*v*_3(1,1,1)_,…, *v*_3(*N*_*x*_,*N*_*y*_,*N*_*z*_)_]^*T*^, and **p** ^*n*^ = [*p*_(1,1,1)_,…, *p*_(*N*_*x*_,*N*_*y*_,*N*_*z*_)_]^*T*^ can be derived:

(13) CyCzDyDzv2n+1v3n+1=−diagCDxC−C˙+μρLC−DxC−1ρDxpn+10.

Here, **p** ^*n*+1^ is the solution to

(14) 1ρLpn+1=−DxDyDzI3⊗diagDDx+diagv2nDy+diagv3nDz·Dv2nv3n,

where **C** = [*𝒟*_(1,1,1)_,…, *𝒟*_(*N*_*x*_,*N*_*y*_,*N*_*z*_)_]^*T*^, $\dot{C}=[{\left(\partial 𝒟/\partial t\right)}_{\left(1,1,1\right)},\ldots ,{\left(\partial 𝒟/\partial t\right)}_{\left(N_{x},N_{y},N_{z}\right)}{]}^{T}$, *ℂ*_*y*_ = diag⁡([(∂*𝒟*/∂*y*)_(1,1,1)_,…, (∂*𝒟*/∂*y*)_(*N*_*x*_,*N*_*y*_,*N*_*z*_)_]), and *ℂ*_*z*_ = diag⁡([(∂*𝒟*/∂*z*)_(1,1,1)_,…, (∂*𝒟*/∂*z*)_(*N*_*x*_,*N*_*y*_,*N*_*z*_)_]). Note that diagonal entries of *ℂ*_*y*_ and *ℂ*_*z*_ in (13) are the derivatives of measured data **D**. Since the linear system in (13) may not be diagonally dominant and lead to severe numerical instability, we consider a minimization problem for **v** ^*n*+1^ = [**v**_2_^*n*+1^, **v**_3_^*n*+1^]^*T*^ with a regularization term as follows:

(15) $$\begin{array}{c} \underset{v^{n+1}}{min}{\left\|\;Sv^{n+1}-f^{n+1}\;\right\|}_{2}^{2}+\lambda^{2}{\left\|\;v^{n+1}-v^{n}\;\right\|}_{L}^{2}, \end{array}$$

where *λ* is the regularization parameter and

(16) S=CyCzDyDz,fn+1=−diag⁡CDxC−C˙+μρLC−DxC−1ρDxpn+10.

The first term in (15) is a penalty term which makes a solution satisfy the proposed reconstruction model, while the second term mitigates the ill-posedness of the diagonal matrices *ℂ*_*y*_ and *ℂ*_*z*_. The minimizer of (15) is given by

(17) $$\begin{array}{c} v^{n+1}=v^{n}+{\left(\;S^{T}S+\lambda^{2}I_{2N}\;\right)}^{-1}\left(\;S^{T}\left(\;f^{n+1}-Sv^{n}\;\right)\;\right), \end{array}$$

where *N* = *N*_*x*_ × *N*_*y*_ × *N*_*z*_. During the iterative procedure, we used a stopping criterion as

(18) $$\begin{array}{c} \frac{{\left\|v^{n+1}-v^{n}\right\|}_{2}}{{\left\|v^{n}\right\|}_{2}}<10^{-2}. \end{array}$$

### 2.3. Initial Guess

For better convergence in solving the minimization problem in (15), we determine a proper initial guess **v** ^0^. For vorticity **ω**≔∇×**v**, we assume that projection of the vorticity in a direction **d** parallel to the imaging plane passing through two valves is negligible:

(19) $$\begin{array}{c} d\cdot \omega \approx 0. \end{array}$$

For computational simplicity, the third component of **d** is set to zero:

(20) $$\begin{array}{c} d=\left(\;d_{1},d_{2},0\;\right). \end{array}$$

Here, *d*_1_ and *d*_2_ are determined by the Doppler data *𝒟* and divergence-free condition. From (19) and (20), we have

(21) 0d1,d2,0·ω=d1∂∂yv3−∂∂zv2−d2∂∂xv3−∂∂zv1=−d1∂∂zv2+d1∂∂yv3−d2∂∂xv3+d2∂∂zv1.

Substituting *v*_1_ = *𝒟* in (21) yields

(22) $$\begin{array}{c} -d_{1}\frac{\partial }{\partial z}v_{2}+\left(\;d_{1}\frac{\partial }{\partial y}-d_{2}\frac{\partial }{\partial x}\;\right)v_{3}\approx -d_{2}\frac{\partial }{\partial z}D. \end{array}$$

We consider two combinations of *y* and *z* directional derivatives of (22) and $\underset{\left(*\right)}{\underset{︸}{the\;\;second\;\;equation\;\;of\;\;(3)}}$ such that −∂/∂*z*(22) + *d*_1_(∂/∂*y*)(*∗*) and ∂/∂*y*(22) + *d*_1_(∂/∂*z*)(*∗*). Then, we have the following two equations:

(23) d1∇yz2v2+d2∂2∂z∂xv3=d2∂2∂z2−∂2∂x∂yD,d1∇yz2−d2∂2∂x∂yv3=−d2∂2∂y∂z−d1∂2∂z∂xD,

with boundary conditions

(24) v2n+1=v2wall,v3n+1=v3wallon  Γtw,∂v2n+1∂n=0,∂v3n+1∂n=0on  Γtu.

Using the discrete operators in Section 2.2, we obtain the following linear system:

(25) d1Lyz−d2DzDxOd1Lyz+d2DxDyv20v30=−d2Lz−DxDyDd2DyDz−d1DzDxD,

where *𝕃*_*yz*_ = (*𝕃*_*N*_*z*__ ⊗ *𝕀*_*N*_*y*__ ⊗ *𝕀*_*N*_*x*__)+(*𝕀*_*N*_*z*__ ⊗ *𝕃*_*N*_*y*__ ⊗ *𝕀*_*N*_*x*__) and *𝕃*_*x*_ = *𝕀*_*N*_*z*__ ⊗ *𝕀*_*N*_*y*__ ⊗ *𝕃*_*N*_*x*__. Therefore, we obtain the initial guess **v** ^0^ = (*v*_2_^0^, *v*_3_^0^) by solving (25).

### 2.4. Estimation of Boundary Condition: LV Contour Segmentation

The proposed reconstruction model in (9) requires time-dependent boundary conditions defined in (10). In this study, for the 3D velocity boundary conditions on the LV wall, time-varying LV boundaries are tracked and local displacements of the LV wall are estimated. Similar to the LV boundary tracking in 3D echocardiographic data [12], we generate 3D LV surface by interpolating planar LV contours tracked on multiple 2D echocardiographic imaging planes, where each 2D LV contour is tracked by LV boundary tracking method on each imaging plane. Among various 2D LV tracking methods, we use the LV tracking method combined with the Lucas-Kanade method and a constraint formulated by the global deformation of nonrigid heart motion proposed in [13]: for each *i*th tracking point **x**_*i*_(*t*) = (*x*_*i*_(*t*), *y*_*i*_(*t*)), we estimate its velocity **v**_*i*_ = *d* **x**_*i*_/*dt* by minimizing

(26) Ftv1,…,vn=12·∑i=1n∇Ixi,1′,tT⋮∇Ixi,k′,tTvi+∂∂tIxi,1′,tT⋮Ixi,k′,tT22+λ~XTX−1XTx1tT+v1tT⋮xntT+vntTT·xi0yi01−xit−vi22,

where **x**_(*i*, 1)_′,…, **x**_(*i*, 1)_′ are neighborhood pixels of **x**_*i*_ and $\tilde{\lambda }$ is the regularization parameter and

(27) $$\begin{array}{c} X=\left[\begin{array}{cc} x_{1}{\left(0\right)}^{T} & 1\\ \vdots & \\ x_{n}{\left(0\right)}^{T} & 1 \end{array}\right]. \end{array}$$

The first term in (26) represents local motions corresponding to the movements of speckle pattern in B-mode image based on Lucas-Kanade method, while the velocity **v**_*i*_ is regularized by the second term. From **x**_*i*_(*t*), for *i* = 1,2,…, *N*, 2D LV contour Γ(*t*)≔{(*x*(*t*, *s*), *y*(*t*, *s*)) : 0 < *s* < 1} at time *t* is interpolated so that

(28) $$\begin{array}{c} \left(\;x\left(\;t,s_{i}\;\right),y\left(\;t,s_{i}\;\right)\;\right)=x_{i}\left(\;t\;\right) \end{array}$$

for 0 = *s*_1_ < *s*_2_ < ⋯<*s*_*N*_ = 1.

### 2.5. Setting for Forward Simulation

**Figure 3:**
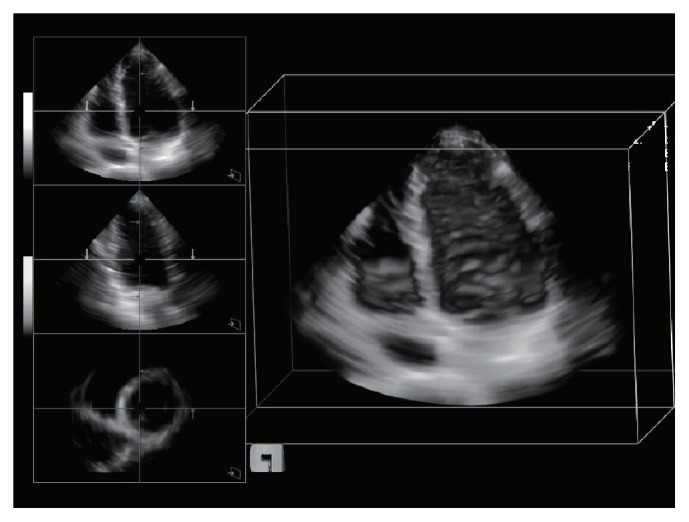
Multiple views of 3D cardiac volume images. The left column shows coronal, sagittal, and axial views in order, and right column represents an image for 3D volume rendering.

For the forward simulation, we use a series of real 3D LV volume data, acquired by a Siemens ACUSON SC2000 imaging system with a 4Z1c probe. The acquisition conditions are given by the transmitting center frequency 2.8 MHz, the mechanical index 0.92, and the thermal index 0.46. The dataset consists of 10 frames within the whole cardiac cycle. The multiple slices are set to sagittal and coronal planes, as shown in Figure 3, and we apply a 2D LV contour tracking method independently of the sagittal and coronal planes. Although ultrasound images including echo and color flow Doppler are obtained from real-time measurements, the scanning time for ultrasound images is bounded below due to dependence on the sound speed. Lately, the development of ultrafast imaging system is ongoing in some research groups. Such a system is expected to have a frame-rate higher than 1,000 fps (for echo images) using plane wave beam-forming techniques. Unfortunately, we do not have such an imaging system yet but have real 3D echocardiography data.

**Figure 4:**
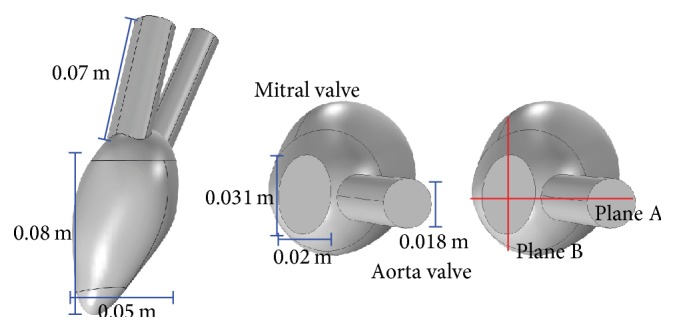
LV model for numerical experiments. The model was reconstructed from ultrasound images.

Figure 4 illustrates 3D LV surface reconstructed by simplifying valve geometry into the pipe-type structure and integrating the LV contours tracked on the two orthogonal imaging planes A and B, which correspond to the coronal and sagittal planes in Figure 3, respectively. In the 3D LV model, the lengths of two pipes corresponding to* mitral* and* aorta* valves are set to be similar to the axial dimension of the LV so that the pressure *p* is developed well from the end of the pipes to the interior of LV region. The cross sections of* mitral* and* aorta* pipes are modeled by elliptical and circular shapes, respectively.

We use commercial software programs for convenience in performing numerical simulations of the forward problem. FreeCAD® is used to integrate the contours into 3D domain at each frame and COMSOL® Multiphysics is used to import the reconstructed 3D domains. The volume of the LV domain and the area of LV wall are then computed at each time step. We denote the variations of volume and area by *V*(*t*) and *A*(*t*), respectively. Here, we consider *dV*(*t*)/*dt*/*A*(*t*) as an average speed $\bar{v}$ at all surface points of the reconstructed LV. The fluid-structure interaction (FSI) model in COMSOL Multiphysics is used to solve (1), assuming that the nodes of the LV boundary move along the outward normal direction **n** with the average speed at each time step. The corresponding velocity **v** at the nodes of the LV boundary are defined as

(29) $$\begin{array}{c} \begin{array}{cc} & v=\bar{v}n\;on\;\;\partial \Omega \setminus \left(\Gamma^{I}\cup \Gamma^{O}\right). \end{array} \end{array}$$

The Neumann boundary condition for pressure is applied at the nodes:

(30) $$\begin{array}{c} \frac{\partial p}{\partial n}=0\;on\;\;\partial \Omega \setminus \left(\Gamma^{I}\cup \Gamma^{O}\right). \end{array}$$

Since the* aorta* valve is closed but the* mitral* valve remains open during the diastole phase, we consider that no viscous stress conditions for **v** with constant pressure are applied at the inlet valve Γ^*I*^, while zero-velocity conditions are applied at the outlet valve Γ^*O*^:

(31) v=0on  ΓO,n·∇v+∇vT=0,p=c1on  ΓI.

During the systole phase, the boundary conditions in (31) are imposed in the opposite way:

(32) v=0on  ΓI,n·∇v+∇vT=0,p=c2on  ΓO.

In this study, we set *ρ* = 1050 kg/m^3^ and *μ* = 0.00316 Pa·s for the density and viscosity of the blood flow, respectively [14]. Stroke volume is about 70 mL, heart rate is 60 per minute [15], and ratio of the diastole to one cardiac cycle is set to 0.6 [16].

## 3. Results and Discussion

### 3.1. Forward Simulation of Blood Flow in LV

**Figure 5:**
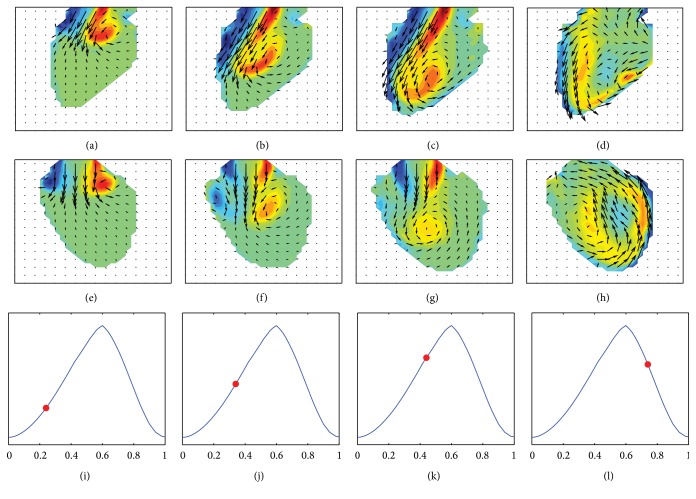
Synthetic intraventricular velocity fields and out-of-plane vorticity component on imaging planes A and B in the first and second rows, respectively, along with volume curves in the third row. On each volume curve, a red dot indicates the corresponding phase for the velocity fields and vorticity contours.

We perform the forward numerical simulation using the FSI model in COMSOL Multiphysics in order to obtain 3D synthetic flow data inside the moving LV domain.  Figure 5 shows synthetic intraventricular velocity and out-of-plane vorticity fields on two orthogonal imaging planes. The velocity and vorticity fields on planes A and B are shown in Figures 5(a)–5(d) and Figures 5(e)–5(h), respectively. The velocity fields are illustrated with velocity vectors, while the colored contours in Figures 5(a)–5(h) represent the out-of-plane vorticity fields. Here, warm (such as red) and cool (such as blue) colors correspond to clockwise and counterclockwise rotating vortex patterns, respectively. In addition, Figures 5(i)–5(l) indicate the time-varying LV volumes marked by a red dot at selected time frames in the cardiac cycle. Since we only consider ten samples from echocardiographic images over one cardiac cycle, the volume curve does not capture early and atrial waves. At the early stage of the diastole phase, Figures 5(a) and 5(e) indicate that two counterclockwise rotating vortices formed near the inlet. Later, the two vortices move toward the apex while maintaining their shape, and the left vortex starts to interact with the lateral LV wall, as shown in Figures 5(b) and 5(f). Due to the interaction, the left clockwise rotating vortex disappears in Figures 5(c) and 5(g), while the counterclockwise rotating vortex occupies the area near the apex and leads to making a larger swirling pattern. In the systole phase, the counterclockwise rotating vortex moves to the right LV wall and the corresponding blood flow heads toward the outlet valve, as shown in Figures 5(d) and 5(h).

### 3.2. Reconstruction of Blood Flow in LV

**Figure 6:**
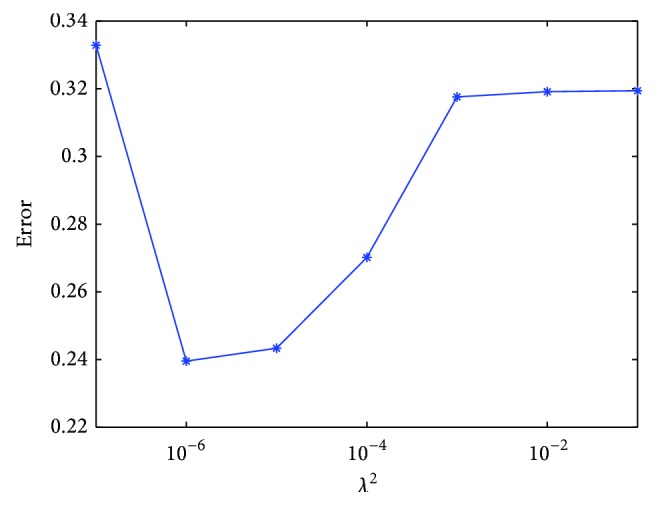
Error change depending on the regularization parameter *λ* ^2^.

Before proceeding further, we investigate the errors of the reconstructed velocity fields that are assessed with respect to the change of *λ* ^2^ from 10^−7^ to 10^−1^ in order to optimize the regularization parameter in (15). The reconstruction error is determined based on *L*_2_-norm error between the reconstructed velocity fields **v** ^*r*^ and the forward data **v** ^*f*^:

(33) $$\begin{array}{c} \frac{\sqrt{\sum_{t=1}^{N_{t}}{\left\|v^{r}-v^{f}\right\|}_{2}^{2}}}{\sqrt{\sum_{t=1}^{N_{t}}{\left\|v^{f}\right\|}_{2}^{2}}}, \end{array}$$

where *N*_*t*_ is the number of time steps.  Figure 6 indicates the minimum error at *λ* ^2^ = 10^−6^, which is used to reconstruct the blood flow in LV.

**Figure 7:**
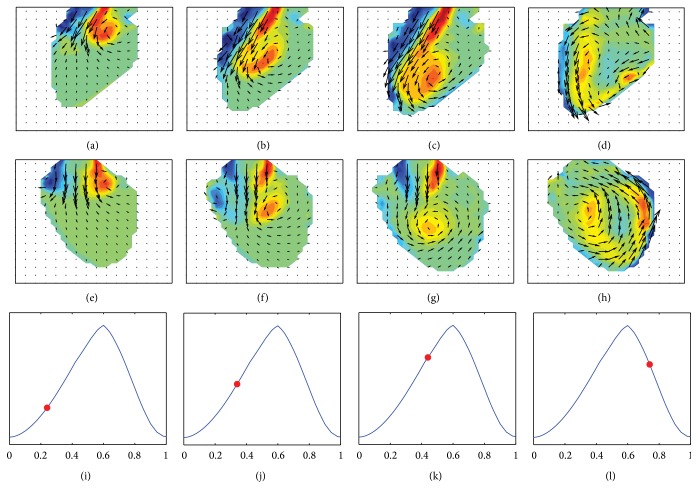
Reconstructed intraventricular velocity and out-of-plane vorticity fields on imaging planes A and B in the first and second rows, respectively, along with volume curves in the third row. On each volume curve, a red dot indicates the corresponding phase for the velocity and vorticity fields.

We consider one-directional velocity component of the synthetic flow data from the forward simulation as color Doppler data. Based on the velocity component, the velocity fields in LV are reconstructed by solving the minimization problem in (15). For better convergence, the initial guess **v** ^0^ is obtained by solving (25) at each time step. The reconstructed solution **v** ^*n*^ is obtained by repeatedly updating the minimizer in (17) until the stopping criterion (18) is satisfied.  Figure 7 illustrates the performance of the proposed reconstruction model. During the diastole phase in Figures 7(i)–7(k), the present reconstruction model clearly captures the counterclockwise rotating flow patterns as well as incoming velocity vector fields heading to the apex, as shown in Figures 7(a)–7(c) and 7(e)–7(g). Moreover, Figures 7(d) and 7(h) clearly show the movement of the counterclockwise rotating vortex to the right LV wall for the systole phase in Figure 7(l). It is worth noting that the proposed model provides accurate reconstructions of the blood flow in a strong vortex region, while the model seems to smooth out small-scale vortex patterns due to the regularization. The maximum magnitude of local velocity error is less than 0.16 m/s and 0.29 m/s during the diastole and systole phases, respectively. Note that the maximum speeds in the diastole and the systole cycle are 0.62 m/s and 1.2 m/s, respectively. This implies that the maximum error scaled by the maximum speed in LV is around 25% during the whole cardiac cycle.

For further quantitative comparison, we investigate reconstruction errors of the velocity and vorticity fields based on the local *L*_2_-norm errors and the differences in the global energy estimation, as listed in Table 1. The local velocity errors are less than 21% during the diastole phase, while the errors are slightly increased up to 32% during the systole phase. In the diastole phase except for the early stage, the local vorticity errors are less than 30%. However, the vorticity errors are increased up to 50% in the rest of the cardiac cycle, because the blood flow in LV exhibits weak vorticity patterns, relative to the diastole phase. Although the local reconstruction errors are not ignorable, the differences of the global energy estimates between the reconstructed flow fields and reference data are less than 7% both for the velocity and for vorticity fields. Thus, the pointwise errors at the local regions may not affect the major features of vortex flows. Furthermore, compared to the previous 2D reconstruction model [11], Table 1 confirms that the proposed 3D model provides better reconstruction performance in terms of local and global errors of the velocity and vorticity fields.

## 4. Conclusions

We proposed a mathematical framework involving a reconstruction model for three-dimensional blood flow inside LV. This framework consists of the extraction of time-varying LV boundaries from multiple echocardiographic images, the forward simulation of the blood flow, and the reconstruction model that combines 3D incompressible Navier-Stokes equations with one-direction velocity component from the synthetic flow data (or color Doppler data) from the forward simulation (or measurement). Assuming that an ultrasound imaging device provides color Doppler data in the whole 3D LV region, we formulated an inverse problem to reconstruct the blood flows, directly embedding the Doppler data in the Navier-Stokes equations and incorporating with the extracted LV boundaries. To generate synthetic Doppler data, we performed the forward simulation of the blood flow using the FSI model and extracted one-directional velocity component of the synthetic flow data. The blood flow inside LV was reconstructed by solving the inverse problem with a proper initial guess of unknown velocity components. Similar to the forward simulation, time-evolving vortex patterns over a cardiac cycle were clearly found in the reconstructed velocity and vorticity fields obtained from the proposed model. We also quantified the reconstruction errors based on the local and global differences between the reconstructed and synthetic flow data. Compared to the previous reconstruction model [11], the proposed model significantly improves the performance of the reconstruction of the blood flow. Through the numerical simulation, we demonstrated the feasibility and potential usefulness of the proposed model in reconstructing the intracardiac blood flows inside the LV.

## Figures and Tables

**Table 1:** Comparison of *L*_2_-norm errors for the velocity field recovered by the proposed reconstruction model and synthetic velocity field. The first and second columns represent normalized pointwise error of velocity and vorticity in *L*_2_, respectively. The third and fourth columns represent normalized *L*_2_-norm in global energy estimates of velocity and vorticity, respectively. Here, $\left\|\right.v{\left.\right\|}_{2}=\sqrt{\sum \left(v_{1}^{2}+v_{2}^{2}+v_{3}^{2}\right)}$, Δ**v** = **v** ^*r*^ − **v** ^*f*^, Δ*k*/*k* ^*f*^ = |‖**v** ^*f*^‖_2_ − ‖**v** ^*r*^‖_2_|/‖**v** ^*f*^‖_2_,  Δ*ω* = *ω* ^*r*^ − *ω* ^*f*^, and Δ*k*_*ω*_/*k*_*ω*_^*f*^ = |‖*ω* ^*f*^‖_2_ − ‖*ω* ^*r*^‖_2_|/‖*ω* ^*f*^‖_2_. Note that the superscripts *f* and *r* denote the forward and reconstructed data, respectively.

*t*/*T*	‖Δ**v**‖_2_/‖**v** ^*f*^‖_2_	‖Δ*ω*‖_2_/‖*ω* ^*f*^‖_2_	Δ*k*/*k* ^*f*^	Δ*k*_*ω*_/*k*_*ω*_^*f*^
0.05	16.7%	62.4%	0.4%	4.1%
0.15	11.8%	20.4%	0.2%	3.0%
0.25	18.7%	29.3%	2.9%	6.2%
0.35	15.9%	26.0%	3.1%	4.8%
0.45	16.8%	27.6%	0.3%	4.5%
0.55	20.9%	30.9%	1.4%	4.9%
0.65	26.8%	38.6%	2.0%	6.7%
0.75	29.9%	45.1%	5.0%	6.9%
0.85	32.2%	50.4%	0.4%	4.1%
0.95	32.2%	48.6%	0.4%	4.1%
